# Utilization of Bacteriophage phi6 for the Production of High-Quality Double-Stranded RNA Molecules

**DOI:** 10.3390/v16010166

**Published:** 2024-01-22

**Authors:** Alesia A. Levanova, Minna M. Poranen

**Affiliations:** Molecular and Integrative Biosciences Research Programme, Faculty of Biological and Environmental Sciences, University of Helsinki, 00014 Helsinki, Finland; minna.poranen@helsinki.fi

**Keywords:** bacteriophage phi6, cystovirus, dsRNA virus, RNA-dependent RNA polymerase, double-stranded RNA production, RNA interference, antiviral siRNA, siRNA pool

## Abstract

Double-stranded RNA (dsRNA) molecules are mediators of RNA interference (RNAi) in eukaryotic cells. RNAi is a conserved mechanism of post-transcriptional silencing of genes cognate to the sequences of the applied dsRNA. RNAi-based therapeutics for the treatment of rare hereditary diseases have recently emerged, and the first sprayable dsRNA biopesticide has been proposed for registration. The range of applications of dsRNA molecules will likely expand in the future. Therefore, cost-effective methods for the efficient large-scale production of high-quality dsRNA are in demand. Conventional approaches to dsRNA production rely on the chemical or enzymatic synthesis of single-stranded (ss)RNA molecules with a subsequent hybridization of complementary strands. However, the yield of properly annealed biologically active dsRNA molecules is low. As an alternative approach, we have developed methods based on components derived from bacteriophage phi6, a dsRNA virus encoding RNA-dependent RNA polymerase (RdRp). Phi6 RdRp can be harnessed for the enzymatic production of high-quality dsRNA molecules. The isolated RdRp efficiently synthesizes dsRNA in vitro on a heterologous ssRNA template of any length and sequence. To scale up dsRNA production, we have developed an in vivo system where phi6 polymerase complexes produce target dsRNA molecules inside *Pseudomonas* cells.

## 1. Introduction

Bacteriophages have numerous biotechnological and potential medical applications since they are harmless to humans, extensively studied, and relatively easy to manipulate. Several enzymes widely used in molecular biology research originate from bacteriophages, including the DNA-dependent RNA polymerase (DdRp) of the T7 bacteriophage, T4 DNA ligase, and phi29 DNA polymerase. Bacteriophage research has also resulted in important biomedical tools, like the phage display system.

*Cystoviridae* is the only recognized family of bacteriophages whose members have a double-stranded (ds)RNA genome and encode an RNA-dependent RNA polymerase (RdRp) capable of replicating the viral dsRNA genome. The best-studied representative of cystoviruses is phi6, which is the first dsRNA bacteriophage to be isolated [1]. The phi6 virion consists of three structural layers [2], and the polymerase activity required for the synthesis of viral dsRNA genome is associated with its innermost layer, the polymerase complex [3]. Protein P2 was recognized as a catalytic subunit of the polymerase complex and is responsible for the synthesis of both plus and minus strands of the phi6 genome [4].

Unlike the RdRps of animal dsRNA viruses, the phi6 RdRp can be produced as an enzymatically active recombinant protein [5]. The purified phi6 RdRp is not dependent on any helper proteins and can efficiently initiate the synthesis of dsRNA on single-stranded (ss)RNA templates de novo, i.e., in a primer-independent manner. Furthermore, phi6 RdRp utilizes a range of heterologous templates of various lengths and sequences and has a high processivity [5]. These properties of phi6 RdRp make it a valuable biotechnological tool for the production of dsRNA molecules for RNA interference (RNAi) applications. 

The term RNAi was initially suggested to describe gene silencing in *Caenorhabditis elegans* induced by long dsRNA molecules [6]. This form of gene silencing occurs post transcriptionally, when small interfering (si)RNA molecules, obtained through the cleavage of dsRNA with the RNaseIII enzyme Dicer, interact with the cognate mRNA, thereby resulting in its degradation and preventing protein translation. RNAi is an evolutionarily conserved mechanism, and, with a few exceptions, the RNAi protein machinery can be found in any eukaryotic organism [7]. RNAi is a widely used research tool to study gene function. In addition to its research potential, numerous practical applications of RNAi have been recognized. 

In mammalian cells, exogenously introduced siRNAs can inhibit viral replication [8], tumor progression [9], and alleviate symptoms of hereditary diseases [10]. Accordingly, a number of siRNA-based therapeutics for rare hereditary diseases have already been approved for medical use [11]. In plants, fungi, and invertebrates, RNAi is an important natural antiviral defense system [12,13,14,15], and laboratory-produced virus-specific dsRNAs applied to plants can interfere with virus infection in a sequence-specific manner (e.g., [16,17,18]). A growing body of evidence demonstrates that dsRNAs can function as plant-protective agents against viruses, fungi, and pests [19,20], and they offer an alternative to chemical pesticides, which may pose risks for environment and human health [21]. Applying dsRNA to plants can be as simple as spraying it. In a proof-of-concept study, long dsRNA molecules sprayed on barley plants were taken up and transported via the plants’ vascular system, inducing systemic protection as demonstrated by the silencing of the target *Fusarium graminearum* genes, even in tissues distant from the spray site [22]. The first sprayable dsRNA biopesticide against Colorado potato beetle is about to be approved for commercial use in the USA under the trademark Calantha [23]. The active component of Calantha is called Ledprona and represents dsRNA molecules targeting proteasome subunit beta 5 mRNA [24].

## 2. Methods of dsRNA Production for RNAi Applications

Currently, there are three main approaches to generate RNA molecules for RNAi purposes: (1) chemical synthesis, (2) enzymatic synthesis of RNA in vitro using recombinant bacteriophage polymerases, and (3) production of RNA in specifically modified microbial cells (in vivo RNA production systems). In chemical synthesis, phosphoramidites are added to each other in a cyclic process occurring in an automated synthesizer on a solid support, such as controlled pore glass [25]. Chemical synthesis guarantees good yields and purity for RNA molecules under 50 nucleotides (nt), whereas, for longer RNAs, its application is impractical and can be justified only for specific reasons, such as, for instance, when modified RNA must be obtained [26]. If a long RNA molecule needs to be chemically synthesized, a range of short RNAs are produced, followed by ligation with T4 ligase [27].

The common method for the enzymatic production of RNA molecules is the in vitro transcription with T7 DdRp or with a similar enzyme from bacteriophage SP6 [26,28]. In this method, T7 DdRp binds to its promoter sequence at the 5′-end of a DNA template (linearized plasmid or DNA fragment produced by means of a polymerase chain reaction, PCR) and initiates RNA synthesis. The elongation terminates when the enzyme runs off the 3′-end of the template [29]. 

To scale up the dsRNA synthesis and reduce its cost, microbial cell-based in vivo dsRNA production systems have been introduced. Typically, bacterial or yeast cells are transformed with a plasmid, in which the target sequence for RNAi is inserted between two convergent T7 promoters. Alternatively, a sequence forming a hairpin upon transcription can be designed and inserted after a single T7 promoter [30].

In the methods described above, only ssRNAs in their sense and antisense orientation are initially produced. The ssRNAs must be hybridized with their cognate molecules in order to obtain dsRNAs. The process of hybridization, however, is not 100% efficient and might result in the poor yield of biologically active dsRNA molecules. Bacteriophage phi6 RdRp operates directly on ssRNA templates to produce perfect dsRNA duplexes. Below, we will present an extensive overview of the phi6 RdRp-based systems for dsRNA production in vitro and in vivo. The development of both systems is rooted in the fundamental investigations of phi6 RdRp and the polymerase complex, conducted by the research groups of Professor Dennis Bamford and Dr. Minna Poranen. Additionally, we will provide a review of collaborative projects that demonstrate the potential application of our technology in the fields of medicine and agriculture. 

## 3. Phi6 RdRp-Based In Vitro System for dsRNA Production

### 3.1. Biochemical and Structural Properties of phi6 RdRp

Phi6 RdRp was one of the first RdRps that could be produced as a recombinant protein in *Escherichia coli*, purified to a high level of homogeneity, and biochemically characterized [5]. This recombinant enzyme, representing a single monomeric subunit, is highly active in RNA replication, i.e., the synthesis of a complementary RNA strand on an ssRNA template [5]. It can also perform semi-conservative transcription on dsRNA templates, although relatively ineffectively [31], and catalyze terminal nucleotidyl transferase reactions [32]. Currently, a number of RdRps from other dsRNA [33,34,35] and ssRNA [36,37,38,39,40] viruses have been biochemically characterized. However, the extensive biochemical, biophysical, and structural studies carried out on phi6 RdRp during the past two decades have made it one of the best characterized viral RdRp [5,31,32,41,42,43,44]. 

The 2 Å resolution X-ray structure of phi6 RdRp revealed a compact, roughly spherical molecule that has a right-hand fold with palm, fingers, and thumb domains [41]. This structural organization is a hallmark of single-subunit nucleic acid polymerases. Phi6 RdRp shares a conserved common structural core of 231 residues, with all the structurally characterized viral RdRps, including the RdRps of negative-sense (−)ssRNA, positive-sense (+)ssRNA, and dsRNA viruses infecting prokaryotic and eukaryotic hosts [45]. The high level of structural conservation indicates a shared evolutionary origin for viral RdRps that is also reflected in their functional properties.

**Figure 1:**
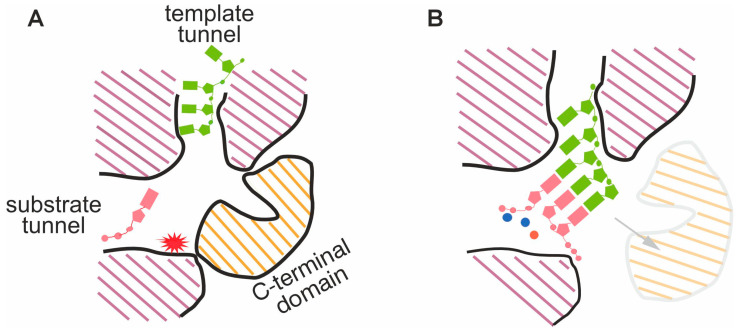
Schematic representation of a phi6 RdRp cross-section. (**A**) The template tunnel is illustrated with the incoming ssRNA template (green). Nucleotides enter through the orthogonally positioned substrate tunnel (pink). The catalytic site, denoted by the red star, is situated in the palm domain. The C-terminal domain harbors a priming platform required for de novo initiation. (**B**) The transition from the initiation mode to the elongation mode of phi6 RdRp activity occurs following the formation of a phosphodiester bond between the first two nucleotides of the daughter strand. Subsequently, the C-terminal domain is likely displaced (shown by arrow), creating an exit tunnel for the synthesized dsRNA molecule. Two Mg^2+^ (depicted as blue circles) and one Mn^2+^ (depicted as a red circle) are involved in the nucleotidyl transfer reaction. The figure is based on [32,41,42,44,46,48].

Inside the spherical phi6 RdRp, a tunnel for template entry into the catalytic site is positioned nearly orthogonally to the tunnel for nucleoside triphosphate entry [41,42] (Figure 1A). The template tunnel as well as its rim is lined with basic amino acids facilitating its interaction with the negatively charged phosphate backbone of incoming RNA. The tunnel is narrow and can only accommodate a single strand of RNA, which is (+)ssRNA in the case of a replication reaction. De novo RNA synthesis is facilitated by the initiation platform in the phi6 RdRp C-terminal region, which stabilizes the enzyme–substrate–template initiation complex [41]. After the switch from initiation to elongation mode, the C-terminal domain is likely displaced to open a path for the exit of dsRNA [41,44] (Figure 1B). The initiation of RNA synthesis occurs at the very 3′ terminus of the ssRNA template [5,46,47], and the resulting dsRNA product is a perfectly duplexed molecule resistant to the RNase I of *E. coli* that readily hydrolyzes ssRNA and partially duplexed dsRNA [5]. The elevated concentrations of purine nucleotides, 1 mM of ATP and GTP versus 0.2 mM of UTP and CTP, substantially enhance dsRNA synthesis [5].

Similar to other known nucleic acid polymerases, the catalysis of nucleotidyl transfer by phi6 RdRp is dependent on divalent metal ions, Mg^2+^ and/or Mn^2+^ [5,49]. Accordingly, two catalytic Mg^2+^ ions have been detected in the high-resolution structure of phi6 RdRp [5,41]. In addition, a third divalent metal ion, Mn^2+^, has been observed in the phi6 RdRp apoenzyme, 6 Å away from the position of the canonical catalytic Mg^2+^ ions [41] (Figure 1B). Interestingly, since then, a similar third metal ion binding site has been described for other viral RdRps [50]. The presence of Mn^2+^ in the reaction buffer is critical for phi6 RdRp-catalyzed RNA synthesis [5,42], especially during the transition from the initiation mode to the elongation one [44]. Ca^2+^ ions replace catalytic Mg^2+^ ions and inhibit RNA synthesis, even in the presence of Mn^2+^, by distorting the geometry of the initiation complex [48]. An isolated phi6 RdRp is a non-specific and highly processive enzyme, which can re-initiate from the same template despite occasional stalling events and, thus, replicate ssRNA at least up to 13.5 kb in length [5,51]. The nucleotide addition rate of phi6 RdRp is not significantly affected by temperature and varies from 16 to 24.6 nt/s in the temperature range between 25 °C and 45 °C [52].

### 3.2. Enzymatic dsRNA Production Using Recombinant phi6 RdRp

**Figure 2:**
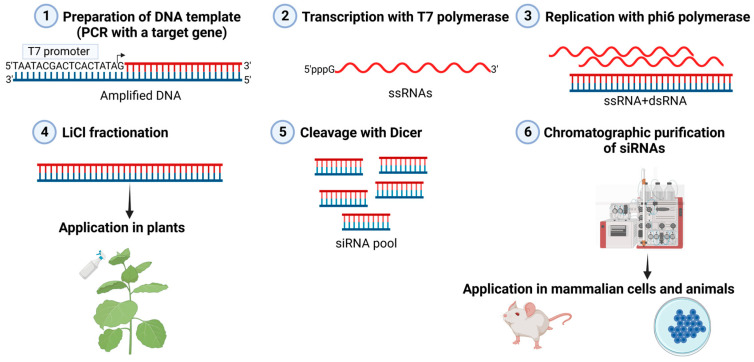
Phi6-based in vitro production of dsRNA molecules. A target gene located downstream of the T7 polymerase promoter is amplified using PCR (**1**), followed by transcription with the DNA-dependent RNA polymerase of bacteriophage T7 (**2**). The resulting single-stranded (ss)RNA contains 5’ triphosphate and is utilized as a template by phi6 RNA-dependent RNA polymerase to produce double-stranded (ds)RNA. (**3**). The dsRNAs are separated from the remaining ssRNA with LiCl fractionation and can be used in plants to induce RNAi (**4**). Alternatively, dsRNA is cleaved with the recombinant Dicer enzyme to generate siRNA pools (**5**). After chromatographic purification, the siRNA pool can be used to induce RNAi in mammalian cells and animals (**6**). The (+) ssRNA is drawn in a red color, and the (−)ssRNA strand is drawn in a blue color.

The simplicity of the production of recombinant phi6 RdRp, the high enzymatic activity of the purified enzyme, and its ability to replicate heterologous ssRNA molecules of practically any sequence and length indicate its potential for dsRNA production [53]. Initially, this production system was established as comprising two sequential reactions. In the first reaction, ssRNA is produced on a DNA template using the DdRp of dsDNA bacteriophage T7, purified, and, subsequently, used as a template for dsRNA synthesis in the second reaction catalyzed by recombinant phi6 RdRp [53]. Alternatively, a single-tube reaction including both polymerases and a DNA template can be set up [54] (Figure 2). This is, normally, a preferable option owing to its simplicity and efficiency. The template for dsRNA production can be either a linearized plasmid or PCR-amplified DNA fragment (Figure 2). Although phi6 RdRp can initiate RNA synthesis virtually on any ssRNA template that has five unpaired nucleotides at the 3′ terminus, corresponding to the distance from the RdRp surface to the catalytic site [43], the sequence at the very 3′ terminus of the ssRNA template does affect the efficacy of the minus strand synthesis [5]. Isolated phi6 RdRp prefers templates that have one or several cytidine residues at the 3′-end [35]. Structural studies on RdRp also indicate that, out of four ribonucleotides, cytidine is preferentially bound into the template’s binding pocket (S pocket) located in the C-terminal domain of the RdRp [41]. 

The phi6-based in vitro platform for dsRNA production is very robust and, depending on the template sequence, generates 30–60 µg of dsRNA from a 2 µg DNA template. Wild-type phi6 RdRp accepts certain modified nucleotides such as, for instance, nucleotides containing fluorine at the 2′ position of the ribose sugar, providing dsRNA with enhanced stability and silencing efficiency [55].

### 3.3. Phi6 RdRp Enables the Production of Long dsRNA Molecules and siRNA Pools for RNAi

Conventionally, RNAi is triggered by one or a few chemically synthetized single-site siRNAs. In such an approach, the choice of a target sequence plays a crucial role in terms of safety and efficiency, especially in antiviral RNAi applications, where the development of escape mutants may risk the long-term use of antiviral siRNAs [56,57]. Many different viral variants circulate simultaneously in host populations making the selection of a single target site in the viral genome rather demanding. Phi6 RdRp allows for the production of long dsRNA molecules, which can be processed into a pool of siRNAs before application to animal cells using the Dicer enzyme or be directly applied to plants, fungi, and insects for subsequent intracellular processing into multiple siRNAs (Figure 2). This process imitates the natural antiviral RNAi pathway in plants and invertebrates, where siRNA molecules target multiple sequences within the viral genome. Under these circumstances, the possibility of mutations in multiple target gene regions resulting in resistance to the siRNA pool becomes negligible. Moreover, each individual siRNA species is highly diluted within the siRNA pools, substantially decreasing the risk of off-target effects. 

The enzymatically produced long dsRNA molecules can be directly applied to plants, followed by their intake and processing by either plant or pathogen RNAi machineries [16,18,22]. In mammalian cells, dsRNA molecules are recognized by a wide range of receptors, including retinoic acid-inducible gene I receptor (RIG-I)-like receptors, protein kinase R, oligoadenylate synthases, and Toll-like receptors, which activate innate immune responses leading to cell growth inhibition and even cell death [58]. This is a protective antiviral mechanism since viral infections are associated with the production of dsRNA molecules. Furthermore, viral polymerases produce RNA molecules carrying triphosphates at the 5′-end (Figure 2), which are also recognized in mammalian cells via RIG-I receptors [59]. To study this phenomenon, Jiang et al. produced a panel of dsRNA molecules using phi6 RdRp and investigated dsRNA-induced innate immune responses in human monocyte-derived dendritic cells [60]. The activation of the innate immune response in dendritic cells was indeed dependent on the dsRNA’s size and 5′-end phosphorylation status [60]. Overall, the results indicated that RIG-I plays a predominant role in the induction of the innate immune responses to dsRNA up to 200 bp, whereas the immunostimulatory activity of longer dsRNAs is likely mediated via another signaling pathway [60]. To reduce the RIG-I-induced activation of interferon-mediated responses, the triphosphate groups from the 5′-ends of the dsRNA molecules produced by viral polymerases can be removed with calf intestinal alkaline phosphatase [28].

To prevent excessive activation of the innate immune system in mammalian cells, enzymatically produced dsRNA molecules are cleaved with the RNase III enzyme Dicer to produce a pool of siRNA molecules (Figure 2). The remnants of undigested dsRNA are removed using anion exchange chromatography or asymmetrical flow field flow fractionation [61,62]. These highly purified siRNA molecules are not toxic and induce only minimal innate immune responses in mammalian cells [54,55,62,63].

Dicer enzymes originating from different organisms produce siRNAs of slightly different sizes, duplexes of 20–25 bp with 2 nt overhangs at the 3′-ends [64,65]. It was reported that longer siRNAs might be beneficial for RNAi in human cells since these siRNA molecules better interact with the human Dicer enzymes that assist their subsequent loading into the RNAi-induced silencing complex, an effector protein complex in the RNAi pathway [66]. The Dicer enzyme from *Giardia intestinalis* generates 27 nt long siRNAs [64,67], which are essentially a substrate for cleavage by human Dicer enzymes. We demonstrated that *Giardia* Dicer-generated siRNAs are safe for mammalian cells and have lower immunostimulatory activity compared to the siRNAs of canonical 22 nt sizes [54] produced with the human Dicer enzymes [68]. The silencing effects were similar for both the human and Giardia Dicer-derived siRNA pools [54].

### 3.4. Antiviral Activity of dsRNA Molecules Produced with phi6 RdRp In Vitro

To date, phi6 RdRp has been utilized for the production of antiviral dsRNA molecules sharing sequence identity with different plant and animal viruses, including the tobacco mosaic virus (TMV), enteroviruses, influenza A viruses (IAV), and herpes simplex virus 1 (HSV1) (Table 1). Niehl et al. [18] utilized phi6 RdRp to produce 2 kbp long anti-TMV dsRNAs targeting the viral replicase gene as well as 0.7 kbp long dsRNA targeting the green florescence protein (GFP) gene inserted in the genome of the GFP-tagged TMV (GFP-TMV) utilized in their study. The dsRNAs applied to *Nicotiana benthamiana* plants infected with GFP-TMV caused a substantial inhibition of virus replication, as suggested by the decrease in GFP fluorescence in the infected plant leaves.

Nygårds et al. [69] applied the human Dicer enzyme to generate a pool of 22 nt long siRNA molecules from a 3.6 kb long dsRNA molecule, which was produced using T7 DdRp, phi6 RdRp, and a complementary (c)DNA template derived from the coxsackievirus B3 genome (Table 1). The pool of purified siRNAs was transfected into monkey kidney LLC-MK2 cells, and the cells were infected with coxsackievirus B3 or the related enteroviruses coxsackievirus B4 and coxsackievirus A9. The antiviral siRNA pool significantly inhibited the replication of all three enteroviruses, because the siRNAs were targeting conservative parts of the enterovirus genomes. Notably, the siRNA pools were three orders of magnitude more efficient than the chemically synthesized single-site siRNA previously used to inhibit coxsackievirus B3 replication [69]. 

We have generated siRNA pools targeting three essential genes of HSV1: *UL27*, *UL29*, and *UL54* [70]. The *UL27* gene encodes the viral envelope glycoprotein B, a class III membrane fusion protein, involved in binding to heparan sulfate [71,72]. The product of *UL29* is an ssDNA-binding protein essential for the replication of HSV genome [73]. The protein encoded by the *UL54* gene is indispensable for viral mRNA biogenesis [74]. The target sequences within each of the HSV1 genes were carefully selected to reduce the probability of off-target effects by excluding significant similarities with mouse or human genomes. The efficiency of these siRNA pools and their equimolar mixture was assessed in three cell lines infected with laboratory and clinical HSV1 isolates [70]. All siRNA pools significantly reduced the replication of all HSV1 strains used in the study, and the effect of UL29 siRNA pool was the most pronounced [70]. The safety, immunostimulatory and antiviral activities of the UL29-specific siRNA pool have been subsequently investigated more extensively in different cell lines representing the tissues where HSV1 replicates and in BALB/c mice (Table 1). The produced UL29 siRNA pool inhibits viral replication in epithelial and nervous tissue-derived cell lines resulting in four orders of magnitude decreases in viral titers [54,70]. Furthermore, the UL29 siRNA pool efficiently reduces symptoms and inhibits viral replication and shedding in a mouse model of corneal HSV1 infection [75]. 

Since phi6 RdRp is capable of incorporating 2′-fluoro-modified nucleotides during the synthesis of dsRNA, we produced UL29 siRNA pools containing modified adenylate, cytidylate, or uridylate in the antisense strand. The antiviral potency of such modified siRNA pools was significantly higher than that of the unmodified UL29 siRNA pool, even though only a fraction of nucleotides was modified [55]. The siRNAs harboring 2′-fluoro modifications in CMP and UMP (2′-F-CMP-siRNA and 2′-F-UMP-siRNA, respectively) elicited substantial innate immune responses in nervous tissue-derived cell lines. However, this immunostimulatory activity did not translate into a higher antiviral potency. Instead, the 2′-F-AMP-siRNAs were well tolerated (showed no cellular toxicity and only slight innate immune responses) in the epithelial tissue-derived cells [55] and in human corneal epithelial cells [76]. The antiviral effects of the 2′-F-AMP-siRNA pools were more pronounced than those of the non-modified siRNA pools, and their inhibition of viral release was close to 100% [76]. Therefore, the enzymatically prepared 2′-F-UL29 siRNA pool is a potential alternative for the treatment of acyclovir-resistant HSV1 eye infections [77]. To further investigate the possibility to treat corneal HSV1 infection with 2′-F-AMP-siRNAs, the siRNA pool was applied in mice, intranasally infected with HSV1. Even a single dose of siRNAs of 750 pmol per mouse administered 4 h post infection inhibited the replication of HSV1 in the orofacial region and protected the mice against the development of severe clinical symptoms [78].

Jiang et al. [79] analyzed the effectiveness of the siRNA pool approach as an antiviral strategy against rapidly evolving viruses. In this study, a chimeric cDNA construct comprising selected conserved genomic sequences of IAV genome was generated and used as a template for the T7 DdRp and phi6 RdRp-directed dsRNA synthesis (Figure 2). The resulting dsRNA was cleaved with the *Giardia* Dicer enzyme to generate an anti-influenza siRNA pool. The produced pool showed a wide-spectrum of anti-influenza A activities and suppressed the replication of human seasonal AIV isolates (H1N1 and H3N2 subtypes) originating from four decades as well as highly pathogenic avian influenza H5N1 and H7N9 viruses (Table 1) in primary human macrophages, monocyte-derived dendritic cells, and lung epithelial Calu-3 and A549 cells [79]. These findings demonstrate the broad-spectrum antiviral potential of siRNA pools and have implications for the use of siRNA pools against infections caused by fast-evolving RNA viruses.

**Table 1:** Antiviral dsRNAs produced with phi6 RdRp.

Virus ^1^	Target Sequences (Size, kb)	Test Organism/Cell Line	References
TMV-GFP	Replicase gene (2), GFP gene (0.7)	*Nicotiana benthamiana*	[18]
Enteroviruses: coxsackievirus B3, B4, A9	2B, 2C, 3A, 3B, 3C, 3D (3.6) ^2^	Monkey kidney LLC-MK2 cells	[69]
HSV1(17+), HSV1(F), HSV1(KOS), clinical HSV1 isolates	*UL27* (0.5), *UL29* (0.7), *UL54* (0.8) ^3^	Human telomerase reverse transcriptase-immortalized retinal pigment epithelial (hTERT-RPE1) cells, human epithelial HaCaT cells, human glioblasma-astrocytoma U373MG cells, immortalized human corneal epithelial (HCE) cells, BALB/c mice	[54,55,63,70,75,76,77,78]
Influenza A virus variants ^4^	Chimeric construct of PB2, PB1, PA, NP, M, NS genes (2.8) ^5^	Human monocyte-derived macrophages and dendritic cells, human lung cancer Calu-3 cells, human lung epithelial A549 cells	[79]

^1^ TMV-GFP, tobacco mosaic virus with green fluorescence protein gene; HSV1, herpes simplex virus type 1. ^2^ Nucleotides 3837–7399 from the Nancy strain of coxsackievirus B3. ^3^ Nucleotides 54689–55207, 59301–59953, and 113947–114715 from the HSV1 strains (17 + ) for *UL27*, *UL29*, and *UL54* genes, respectively. ^4^ Avian A/Vietnam/1194/04 (H5N1) and A/Anhui/1/13 (H7N9); and human A/Udorn/307/1972 (H3N2), A/Beijing/353/1989 (H3N2), A/Wisconsin/67/2005 (H3N2), A/New Caledonia/20/1999 (H1N1), A/Brisbane/59/2007 (H1N1), A/Finland/643/2009 (H1N1). ^5^ Chimeric construct comprising highly conserved sequences of key viral genes based on the A/wild duck/Hunan/211/2005 virus sequences.

Thus, multiple studies conducted on cell lines and laboratory mice have demonstrated the safety and effectiveness of siRNA pools generated through Dicer cleavage of dsRNA produced with T7 DdRp and phi6 RdRp. The capability of phi6 RdRp to incorporate 2′-fluoro-modified nucleotides offers an opportunity to produce 2′-F-siRNAs, surpassing non-modified siRNAs in antiviral activity, and presents a potential means for the development of antiviral therapeutics. Furthermore, siRNA pools have been shown to be effective against various virus variants [70,79], making them a superior alternative to single-site siRNA.

## 4. Large-Scale Production of dsRNA in Pseudomonas Cells

### 4.1. DsRNA Amplification within phi6 Polymerase Complexes

**Figure 3:**
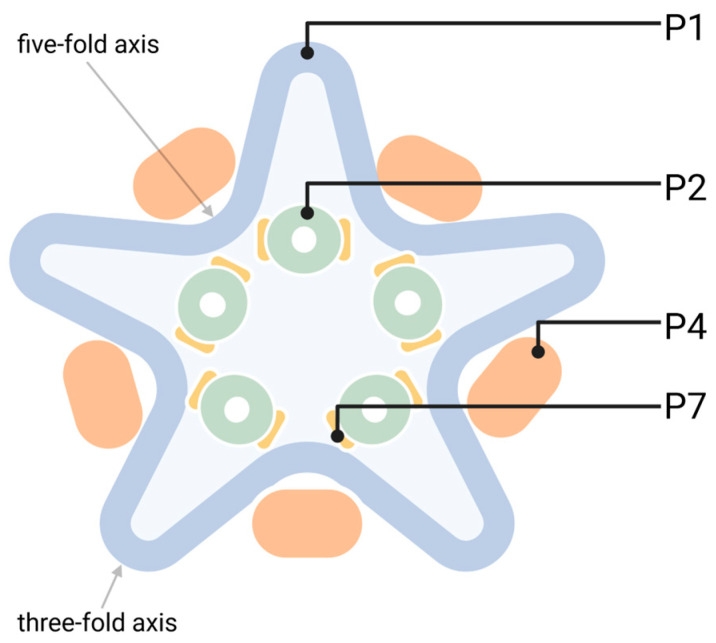
The structural arrangement of the empty polymerase complex (procapsid) of bacteriophage phi6. The dodecahedral polymerase complex is spontaneously assembled from 120 copies of the major inner capsid protein P1. Inside the empty procapsid, P2 RdRps are located close to the three-fold symmetry axes, making contacts with two neighboring five-fold vertices, orientated inwards in the empty polymerase complexes [84,89]. Assembly co-factor P7 is likely located near P2 [83,85]. Packaging NTPase P4 hexamers are located at the recessed icosahedral five-fold vertices, resulting in a symmetry mismatch. The size of the empty polymerase complex is about 46 nm [80]. The protein location is indicated according to [83,84,85,90,91].

During natural phi6 infection in a *Pseudomonas syringae* host [1], the viral dsRNA genome is replicated and transcribed within a proteinaceous capsid, the polymerase complex (Figure 3) which forms the innermost structural layer of the phi6 virion. The structural framework of the polymerase complex is made of 120 copies of the main inner capsid protein P1 arranged on an *T = 1* icosahedral lattice [80,81]. The RdRp P2 and the assembly co-factor P7 [82] are located inside the P1 shell near the three-fold axes of symmetry in an empty polymerase complex, akaprocapsid [83,84,85]. The energy for phi6 ssRNA packaging into procapsids is provided by hexameric NTPase P4, located on the icosahedral vertices of the polymerase complex [86] (Figure 3). In addition, P4 hexamers likely form exit sites for transcripts produced inside the particle [87]. P7 promotes the assembly of the procapsids but is also essential for efficient ssRNA packaging into the particle [82,88].

During natural phi6 infection, the two outer layers of the virion, namely, the envelope and the nucleocapsid surface shell, are shed off, and the phi6 polymerase complex becomes transcriptionally active [92,93]. Within the polymerase complex, P2 produces phi6-specific ssRNAs of positive polarity (l+, m+, and s+). The delivery of the (+)ssRNAs to the cytoplasm is dependent on P4 hexamers on the polymerase complex’s surface [87]. The proteins P1, P2, P4, and P7 are translated from the l+ ssRNA and assembled into new procapsids (Figure 3), into which the phi6-specific (+)ssRNAs are subsequently packaged [94]. The three ssRNA are preferentially packaged in the order of s+, m+, and l+, and the packaging of l+ is the signal leading to the activation of the P2 RdRp and the initiation of the synthesis of complementary RNA strands [95,96,97,98]. The produced tri-segmented dsRNA genome is arranged as a single, tightly packed spool of five concentric layers [99]. During genome packaging and replication, the polymerase complex undergoes structural changes and obtains its mature rounded conformation of about 50 nm [80,81,100]. After the completion of RNA replication, the P2 RdRp initiates transcription using the produced dsRNAs as templates. The transcription is triggered when the 5′-end sequence of the l+ has been replicated [101].

The unique *pac* sequences located at the 5′-ends of the (+)ssRNAs mediate the recognition of the viral RNA for packaging, and each (+)ssRNA segment contains a distinct *pac* sequence folded into a unique stem–loop structure [102]. The packaging initiates from the 5′-end of each segment, and its RNA fold may affect the rate and efficiency of RNA encapsidation [103,104]. Normally, only one full-length copy of each of the three genome segments (6374 bp L-, 4063 bp M-, and 2948 bp S-segment) is packaged inside the phi6 polymerase complex [105]. However, truncated phi6 transcripts containing an intact *pac* sequence can be packaged in multiple copies [106]. 

In contrast to the isolated phi6 RdRp subunit, which can efficiently use different heterologous templates for minus-strand RNA synthesis [5,107], intra-capsid RNA replication by phi6 RdRp is very selective. All three plus strands of the phi6 genome have specific secondary structures near the 3′-ends, with a considerable amount of similarity. These secondary structures protect the RNA against nuclease attacks and are required for efficient intra-capsid RNA replication [108]. Removal of the 3′ end stem–loop structure promotes non-homologous recombination, which, otherwise, is rare in cystoviruses [105]. This type of recombination occurs via template switching and does not require sequence identity at a crossover point [109]. In addition to a specific fold, the sequence at the 3′-end is important for the efficient synthesis of the (−)ssRNA strand. The natural sequence at the 3′-ends of all phi6 (+)ssRNA molecules is 5’-CUCUCUCUCU-3’, and, for instance, sequence 5’-GAGCAGC-3’ and some others do not support intra-capsid minus strand synthesis [105]. However, a replication-deficient (+)ssRNA may be rescued via non-homologous recombination [109]. 

In conclusion, for the efficient production of dsRNA in *Pseudomonas* cells, the assembly of a functional phi6 polymerase complex is essential. Additionally, the RNA to be produced must contain functional phi6 packaging and replication sequences to ensure the proper packaging of (+)ssRNA molecules inside the viral capsid and the subsequent initiation of (−)ssRNA synthesis. Furthermore, the size of the RNA may affect the genetic stability and stoichiometry of the RNA molecules encapsidated and amplified by the phi6 polymerase complex.

### 4.2. In Vivo dsRNA Production Platform Based on phi6 Polymerase Complex Activity

**Figure 4:**
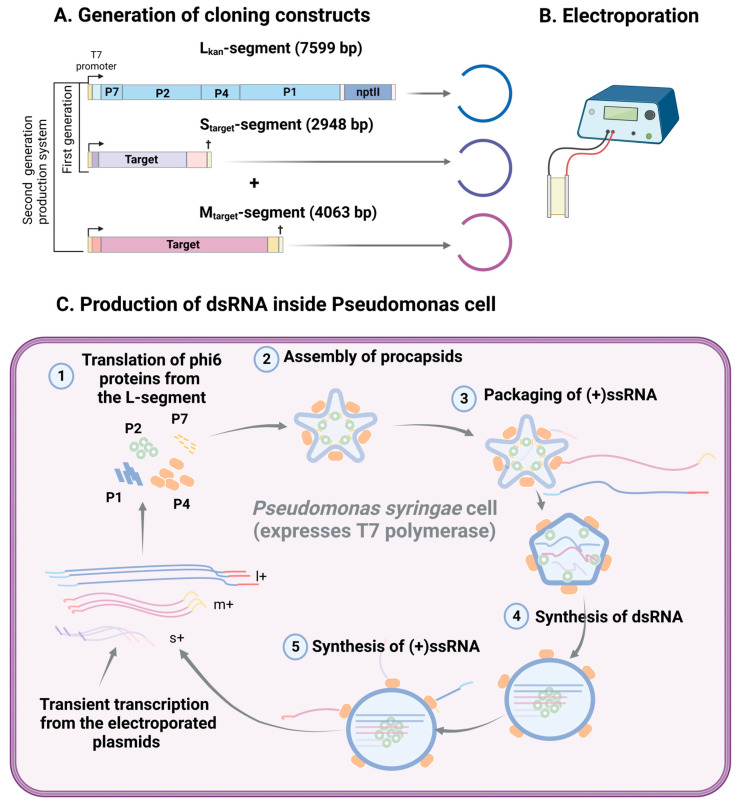
Phi6-based in vivo system for dsRNA production. The target dsRNA sequences are produced as a result of the activity of phi6 polymerase complexes amplifying inside *Pseudomonas syringae* cells. (**A**,**B**) The L-segment of phi6 bacteriophage encodes all the genes required for the production of proteins constituting the polymerase complexes. Therefore, the full-length L sequence is retained in the dsRNA production system. For the selection purposes, the kanamycin resistance gene (*nptII*) is cloned into its 3′ untranslated region (3′ UTR). The phi6 genes on the S- and M-segments are replaced with the target sequences, and only the segment-specific 5′ UTRs and 3′ UTRs, essential for RNA packaging inside the polymerase complex and replication of the encapsidated RNA, are included in the S- and M-segment-specific constructs. All the constructs are under the control of the T7 promoter (shown as a bent arrow) to initiate transcription from the plasmids electroporated into bacteria expressing T7 DdRp. Additionally, the S- and M-segment-specific constructs contain T7 terminator sequences (shown as a cross) downstream the replication signals to ensure dsRNA generation of a correct length. In the first-generation system, only phi6 L- and S-segment-specific constructs are used. To enhance the stability of the dsRNA production system, M-segment sequences are included in the second generation of the dsRNA production system. (**C**) In vivo dsRNA production starts from the transcription directed by T7 polymerase, which is constitutively expressed by the *Pseudomonas syringae* strain. This is a transient step in the formation of the dsRNA production cell line as the plasmids do not replicate in *Pseudomonas* and are, therefore, present only in the initial cell where the plasmids were transformed. The resulting l+ ssRNA molecules serve as messenger RNAs to ensure the production of the P1, P2, P4, and P7 proteins (Step 1), from which polymerase complexes are assembled (Step 2). The empty polymerase complexes are filled with the (+)ssRNA molecules in a sequential order, s+→m+→l+ (Step 3), followed by the replication of the (−)ssRNA strands (Step 4). When the production of dsRNAs has been completed, phi6 RdRp switches into transcription mode to generate new (+)ssRNA molecules, which are extruded into the cytoplasm (Step 5). New polymerase complexes are produced from the proteins translated from the newly synthetized l+ ssRNA, resulting in the accumulation of polymerase complexes and establishment of dsRNA amplification in the transformed bacterium and its daughter cells.

The in vivo dsRNA production system is based on *Pseudomonas syringae* cells carrying phi6 polymerase complexes, in which replication of target dsRNA species occurs (Figure 4). The empty polymerase complexes (Figure 3) are spontaneously assembled in bacterial cells electroporated with a plasmid carrying a cDNA copy of the phi6 L genomic segment, which encodes all the component proteins of the phi6 procapsid [110]. The assembled procapsids support the packaging of the (+)ssRNA molecules carrying phi6-specific *pac* sequences, the synthesis of the (-)ssRNA strands to generate dsRNAs, and the initiation of (+)ssRNA synthesis on newly synthesized dsRNA segments [3,96].

In a first-generation phi6-based in vivo dsRNA production system (Figure 4), two plasmids are electroporated into *Pseudomonas syringae* Cit7 cells, which constitutively express T7 DdRp from a broad host range plasmid, pLM1086 [53]. One of the electroporated plasmid contains a full-length cDNA copy of the phi6 L-segment under a T7 polymerase promoter to facilitate the assembly of active polymerase complexes. A kanamycin resistance gene, derived from transposon Tn903, is inserted at the 3′ UTR of the L-segment [111] to simplify the selection of cells in which dsRNA amplification has been established. The second plasmid contains the sequence of interest flanked by phi6 S-segment-specific packaging and replication sequences at the 5′- and 3′-ends, respectively [53]. These plasmids do not replicate in *Pseudomonas*, and, therefore, resistance to kanamycin is solely dependent on the establishment of RNA amplification by functional phi6 polymerase complexes. In a proof-of-concept study, a 0.7 kb eGFP gene was inserted between the phi6 S-segment-specific packaging and replication sequences. The resulting plasmid was subsequently electroporated into the *P. syringae* cit7 strain, along with the plasmid harboring the full-length cDNA copy of the phi6 L-segment, to establish a *Pseudomonas* clone capable of producing an eGFP-specific dsRNA sequence. In accordance with the previous observation regarding the packaging of multiple copies of segments reduced in size [106], the packaging of three copies of the eGFP S-segment and one copy of the L-segment was observed [53]. 

The major technical problem of the first-generation in vivo dsRNA production system was the high genetic instability of the produced cell lines [18,53]. It seems that the regulatory sequences within the M-segment are essential for stable genome packaging and replication. Furthermore, the addition of the M-segment not only enhances genetic stability but also substantially increases the amount of target-specific dsRNA that can be produced by a phi6 polymerase complex [18]. The second generation of the *Pseudomonas* cell-based system for dsRNA production (Figure 4) includes all three phi6 genome segments, and the sizes of the segments roughly follow the sizes of the wild-type phi6 genome segments. This translates into a higher stability of the dsRNA production system and a lower recombination frequency. The resulting *Pseudomonas* clones contain equimolar quantities of all three dsRNA segments and produce about 700 µg of dsRNA per 100 mL culture (4 × 10^11^ cells) [18]. However, production efficiency might depend on the length and sequence of the insert.

### 4.3. Application of the In Vivo dsRNA Production System for the Synthesis of High-Quality dsRNA for Plant Protection

The phi6-based in vivo dsRNA production platform enables the generation of dsRNA in high quantities, which is essential for agricultural RNAi applications. In a proof-of-concept study by Niehl et al. [18], TMV-specific sequences inserted in phi6 S- and M-segments were amplified in *Pseudomonas* using phi6 polymerase complexes. The amplified sequences were derived from the TMV replicase and movement protein genes. The in vivo produced and purified TMV-specific dsRNAs were applied either mechanically or by spraying to virus-inoculated leaves, and they provided efficient protection against the local and systemic spread of TMV. This protective effect was observed for at least 7 days [18]. 

## 5. Conclusions and Future Research Directions

The enzymatic activity of the phi6 RdRp can be employed for the generation of perfectly duplexed high-quality dsRNA through two approaches: (1) in vitro, using a combination of T7 DdRp and phi6 RdRp; and (2) in vivo, using an RNA replication system based on *Pseudomonas* cells containing phi6 polymerase complexes. Both approaches allow for the production of long dsRNA molecules, which can be subsequently cleaved into a pool of siRNAs either in vitro by recombinant Dicer enzymes or in vivo through the activity of the Dicer enzymes of the target organisms. This process is similar to the naturally occurring RNAi-based antiviral response in plants, fungi, and most invertebrates [12,13]. The use of long dsRNA sequences and siRNA pools makes obsolete the tiresome design of active single-site siRNAs for effective RNAi, reducing the risk of off-target effects and generation of escape mutants.

The enzymatic in vitro dsRNA production system is easily adaptable to sequences of different sizes and nucleotide composition. The sequences for an in vivo dsRNA production system should not divert significantly from the length of the original phi6 segments and should not contain strong hairpin structures impairing ssRNA packaging and, hence, its subsequent replication. Interestingly, polymerase complexes can effectively accommodate dsRNAs that are slightly longer than the original phi6 genome segments [18]. At the same time, attempts to produce dsRNA smaller than the original phi6 genome resulted in the genetic instability of the generatedbacterial clones. Therefore, the dependence of the stability and efficiency of an in vivo dsRNA production system on the length of the target dsRNA molecules is worth further investigation.

The production of dsRNA molecules in Pseudomonas cells can be scaled up to meet the requirements dictated by field applications for plant protection. The phi6 polymerase complex can be programmed to produce dsRNA of two different sequences targeting different genes of the same organism or different pathogens. Once established, a bacterial producing strain is very stable and can be stored at −80 °C for an unlimited period of time. Currently, we purify dsRNA for plant applications with a phenol–chloroform extraction, followed by LiCl fractionation to remove ssRNAs, which is a laborious and expensive method, not suitable for mass-production. Therefore, we need to develop a cost-effective and easy procedure to purify dsRNA. An additional limitation of the in vivo dsRNA production system is the presence of the phi6 L- dsRNA segment, which is not easily separated from the other segments and hampers the estimations of the amounts of active dsRNA molecules. Therefore, it would be beneficial to genetically modify the *Pseudomonas* host and incorporate all the genes required for polymerase complex production into the bacterial genome.

## Data Availability

Not applicable.
